# Distant Cutaneous Metastasis as the First Sign of Recurrence After R0 Resection of Intrahepatic Cholangiocarcinoma: A Case Report

**DOI:** 10.7759/cureus.116987

**Published:** 2026-09-27

**Authors:** Yusuke Takeshita, Kazunori Nojiri, Naoki Maeda, Hidenobu Masui, Takafumi Kumamoto

**Affiliations:** 1 Department of Surgery, Yokosuka Kyosai Hospital, Yokosuka, JPN; 2 Department of Gastroenterological Surgery, Yokohama City University, Yokohama, JPN; 3 Department of Gastrointestinal Surgery, Yokosuka Kyosai Hospital, Yokosuka, JPN

**Keywords:** intrahepatic cholangiocarcinoma, isolated cutaneous metastasis, metastatic cutaneous tumor, non-procedure-related cutaneous metastasis, postoperative subcutaneous nodule

## Abstract

Cutaneous metastasis from cholangiocarcinoma is rare and typically occurs at surgical incision sites or along drainage tracts due to tumor implantation, whereas distant cutaneous metastasis unrelated to surgical procedures is extremely uncommon and generally indicates a poor prognosis. We report the case of a 65-year-old man who was diagnosed with intrahepatic cholangiocarcinoma after a low-density lesion was detected in the left lateral liver segment during postoperative surveillance following nephrectomy for renal cell carcinoma. He underwent left hepatectomy with caudate lobectomy and hepatoduodenal ligament lymphadenectomy, and pathological examination revealed pT2N0M0, stage II disease (AJCC 8th edition). No adjuvant chemotherapy was administered. Eight months after surgery, a painless, mobile subcutaneous mass appeared on the right posterior trunk and was clinically diagnosed as a lipoma. At 14 months postoperatively, the lesion became painful, and partial excision at another hospital revealed a metastatic cutaneous tumor. Subsequent FDG-PET/CT demonstrated uptake in the cutaneous lesion as well as in the right iliac and left femoral bones. Immunohistochemical findings supported cutaneous metastasis from intrahepatic cholangiocarcinoma. The patient received radiotherapy for bone metastasis and systemic chemotherapy, but despite temporary disease control, his condition gradually deteriorated, and he died 56 months after surgery. This case highlights that distant cutaneous metastasis from intrahepatic cholangiocarcinoma may initially present as a benign-appearing postoperative subcutaneous mass and can be easily overlooked. Because cutaneous lesions are readily accessible for diagnostic evaluation, newly developed postoperative subcutaneous masses should be carefully assessed even when serum tumor markers and imaging studies show no evidence of recurrence.

## Introduction

Cutaneous metastasis accounts for approximately 2% of all skin tumors and may occur in up to 10.5% of patients with advanced malignancies [1,2]. In a clinicopathologic study, cutaneous metastasis served as the presenting sign of an underlying cancer in 7.8% of cases, and the median interval from primary tumor diagnosis to cutaneous metastasis was 33 months. The median survival after diagnosis of cutaneous metastasis was only 7.5 months, underscoring its poor prognosis [3].

Intrahepatic cholangiocarcinoma (iCCA), a primary malignancy arising from the intrahepatic bile duct epithelium, is characterized by a high risk of recurrence after curative-intent resection. Hu et al. reported recurrence in 607 of 920 patients (66.0%) who underwent curative-intent resection for iCCA. Among patients with recurrence, 53.2% developed intrahepatic-only recurrence, whereas 46.8% had extrahepatic recurrence, either alone or in combination with intrahepatic recurrence. The lung was the most common extrahepatic site (n=55), followed by lymph nodes (n=46), peritoneum (n=34), and bone (n=13) [4].

Cutaneous metastasis, however, is not a typical pattern of recurrence in iCCA. In the series reported by Lookingbill et al., biliary tract cancers accounted for approximately 4.2% of cutaneous metastases [2]. Most reported cases arise at procedural sites, especially along percutaneous transhepatic biliary drainage tracts or surgical scars [5,6]. In contrast, distant cutaneous metastasis unrelated to procedural routes is exceedingly uncommon and may not be readily recognized as recurrence during postoperative surveillance. 

We report a case of iCCA in which a distant, non-procedure-related cutaneous metastasis led to the diagnosis of systemic recurrence, highlighting the importance of evaluating new postoperative subcutaneous lesions during follow-up.

## Case presentation

A 65-year-old man with a history of right nephrectomy for renal cell carcinoma (RCC) was under routine surveillance. Follow-up computed tomography (CT) revealed intrahepatic bile duct dilatation, and he was referred to our department for further evaluation.

Contrast-enhanced CT demonstrated a 25-mm low-density area to the left of the umbilical portion of the portal vein, and iCCA could not be excluded (Figure 1). The preoperative diagnosis was suspected iCCA, cT2N0M0, clinical stage II (AJCC 8th edition) [7].

**Figure 1:**
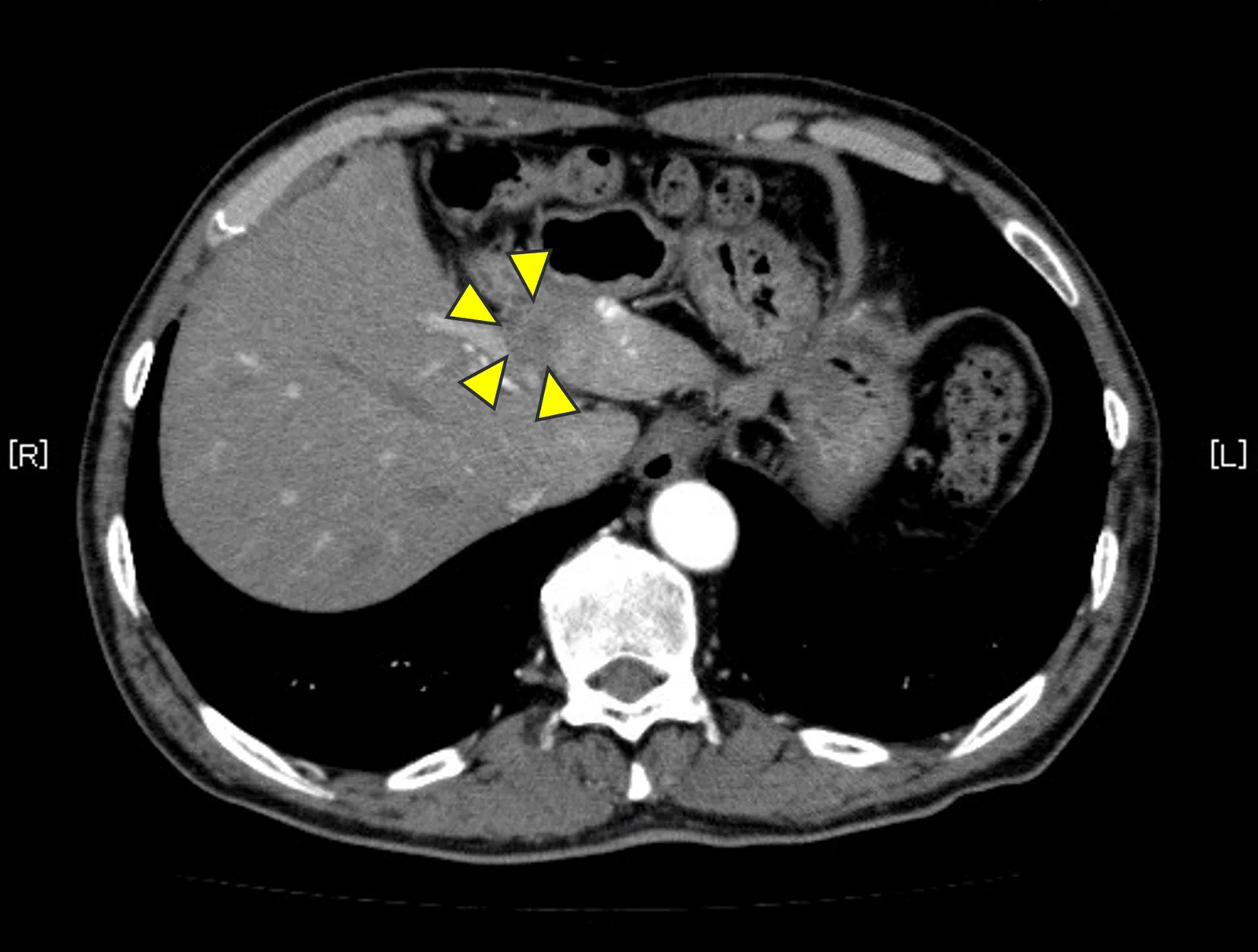
Contrast-enhanced CT of the primary hepatic tumor. Contrast-enhanced CT shows a 25‑mm low‑density area to the left of the umbilical portion of the portal vein (yellow arrowheads).

Left hepatectomy with caudate lobectomy and hepatoduodenal ligament lymphadenectomy was performed, achieving R0 resection.

Histopathology showed moderately to poorly differentiated adenocarcinoma. Immunohistochemistry was CK7-positive and CK20-negative, consistent with iCCA (Figure 2). The final pathological stage was pT2N0M0, stage II (AJCC 8th edition) [7]. At that time, adjuvant chemotherapy was not recommended; therefore, the patient was followed with surveillance without postoperative adjuvant chemotherapy.

**Figure 2:**
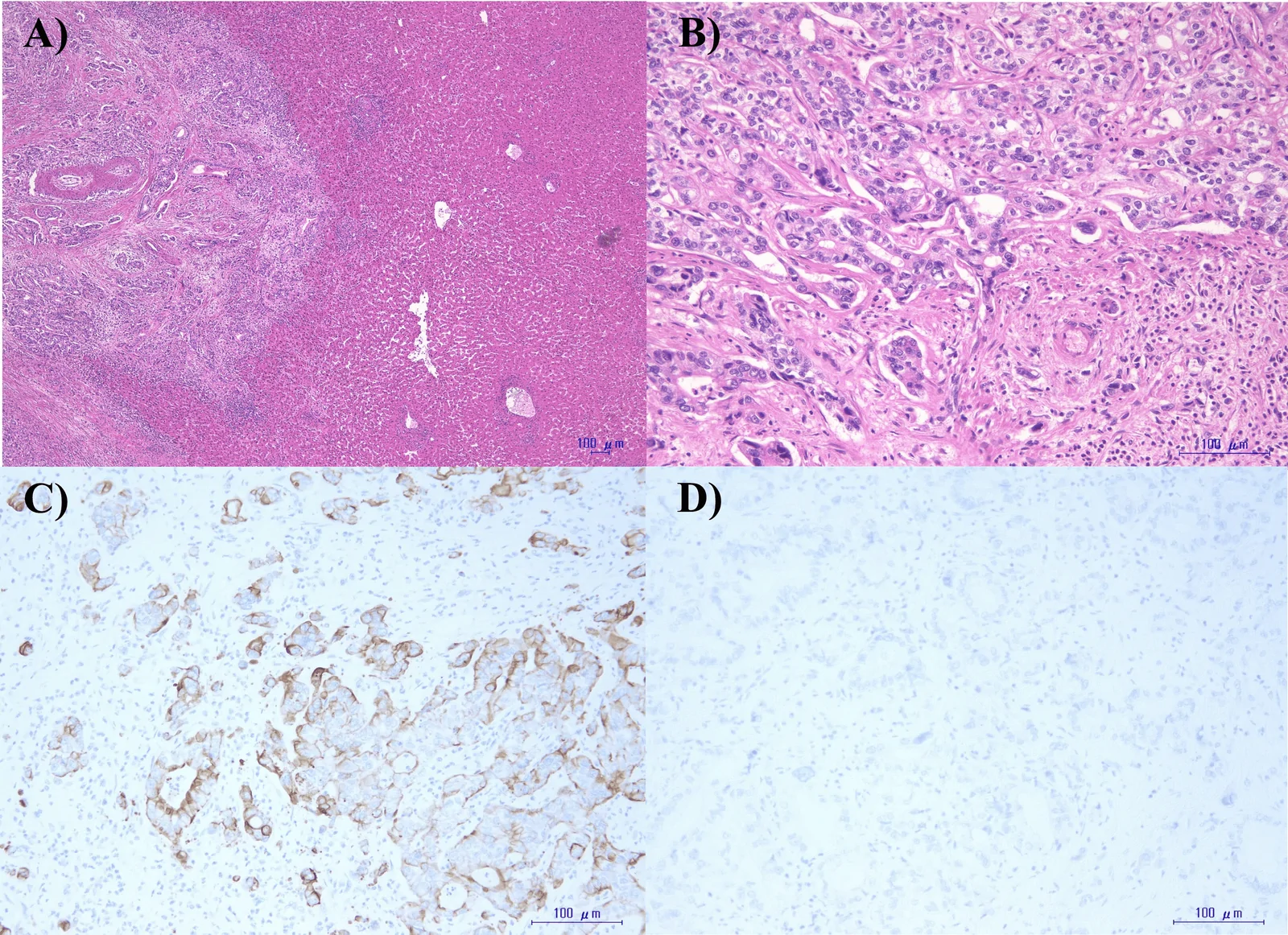
Histopathology of the primary tumor. Hematoxylin and eosin (H&E) staining of the liver revealed moderately to poorly differentiated adenocarcinoma. Immunohistochemistry demonstrates CK7 positivity and CK20 negativity. (A, B) H&E, low- and high-power views; (C) CK7; (D) CK20. Original magnification: (A) ×40; (B–D) ×200.

During outpatient follow-up, the patient noticed a subcutaneous mass on the right posterior trunk at approximately eight months postoperatively but first reported it at the 10-month visit. The lesion was located near the posterior aspect of the right axilla and was a painless, mobile subcutaneous nodule on palpation. A benign lesion such as lipoma was suspected. Serum tumor markers were within normal limits (carcinoembryonic antigen (CEA): 4.0 ng/mL (normal <5.0 ng/mL); carbohydrate antigen 19-9 (CA19-9): 19.1 U/mL (normal <37 U/mL)), and observation was selected. At the 12-month visit, there was no obvious change in the lesion.

At 14 months postoperatively, the lesion became painful, and partial excision was performed at another hospital. Pathology indicated a metastatic cutaneous tumor, and the patient was referred to our dermatology department.

FDG-PET/CT performed for staging demonstrated uptake in the cutaneous lesion (SUVmax 4.7) as well as abnormal uptake in the right iliac bone (SUVmax 3.6) and left femur (SUVmax 7.0) (Figure 3). Serum markers immediately before PET/CT remained within reference ranges (CEA: 3.8 ng/mL (normal <5.0 ng/mL); CA19-9: 34.4 U/mL (normal <37 U/mL)).

**Figure 3:**
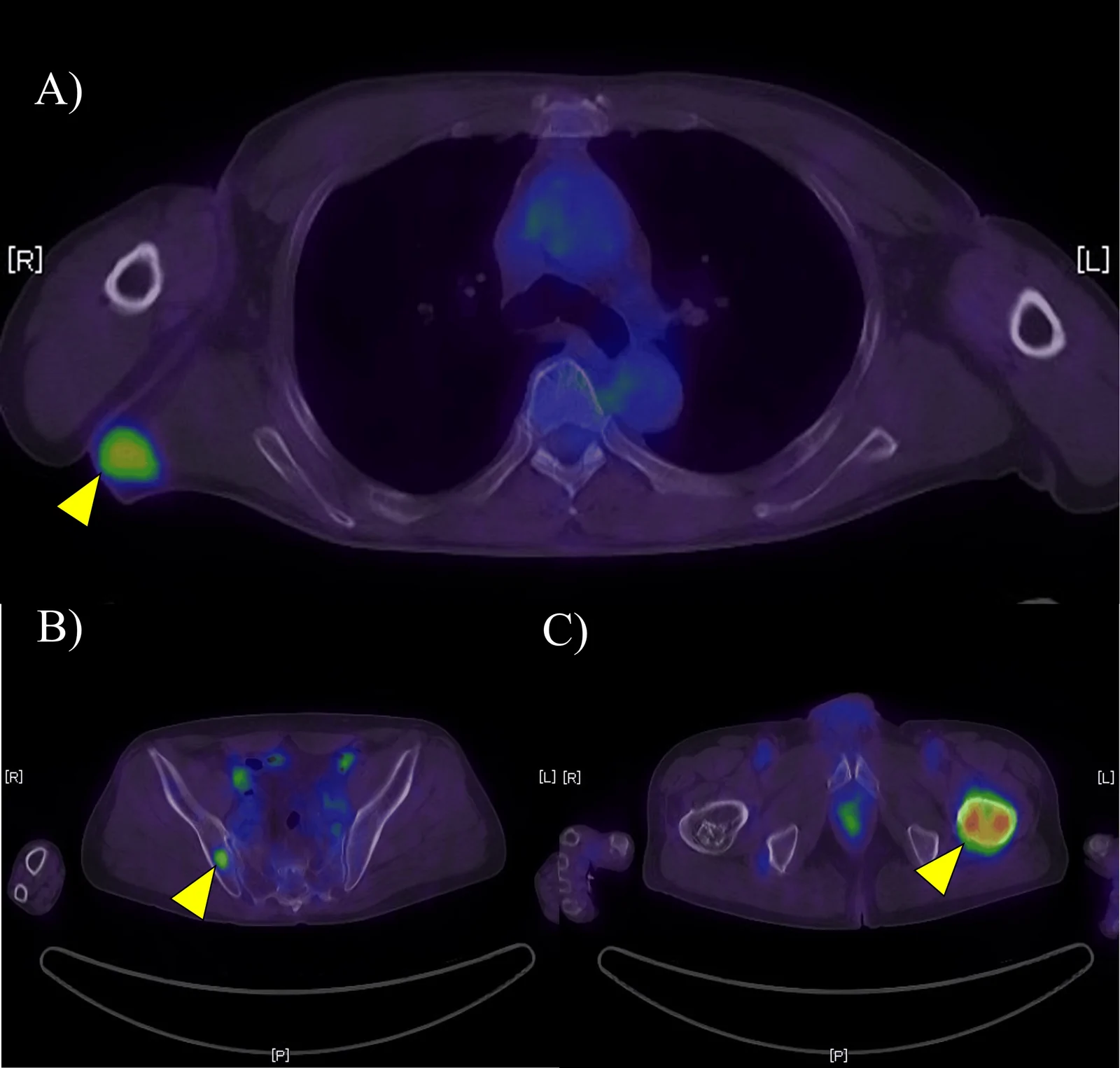
FDG-PET/CT at recurrence. (A) FDG uptake in the cutaneous lesion. (B) Uptake in the right iliac bone. (C) Uptake in the left femur, consistent with metastases.

Given the patient’s history of RCC, metastatic RCC was considered. The prior RCC was CK7-negative/CK20-negative and PAX8-positive, whereas the cutaneous lesion was CK7-positive/CK20-negative and PAX8-negative (Figure 4). Additional immunohistochemical evaluation of the archived primary iCCA specimen also showed PAX8 negativity, supporting recurrence from iCCA rather than RCC.

**Figure 4:**
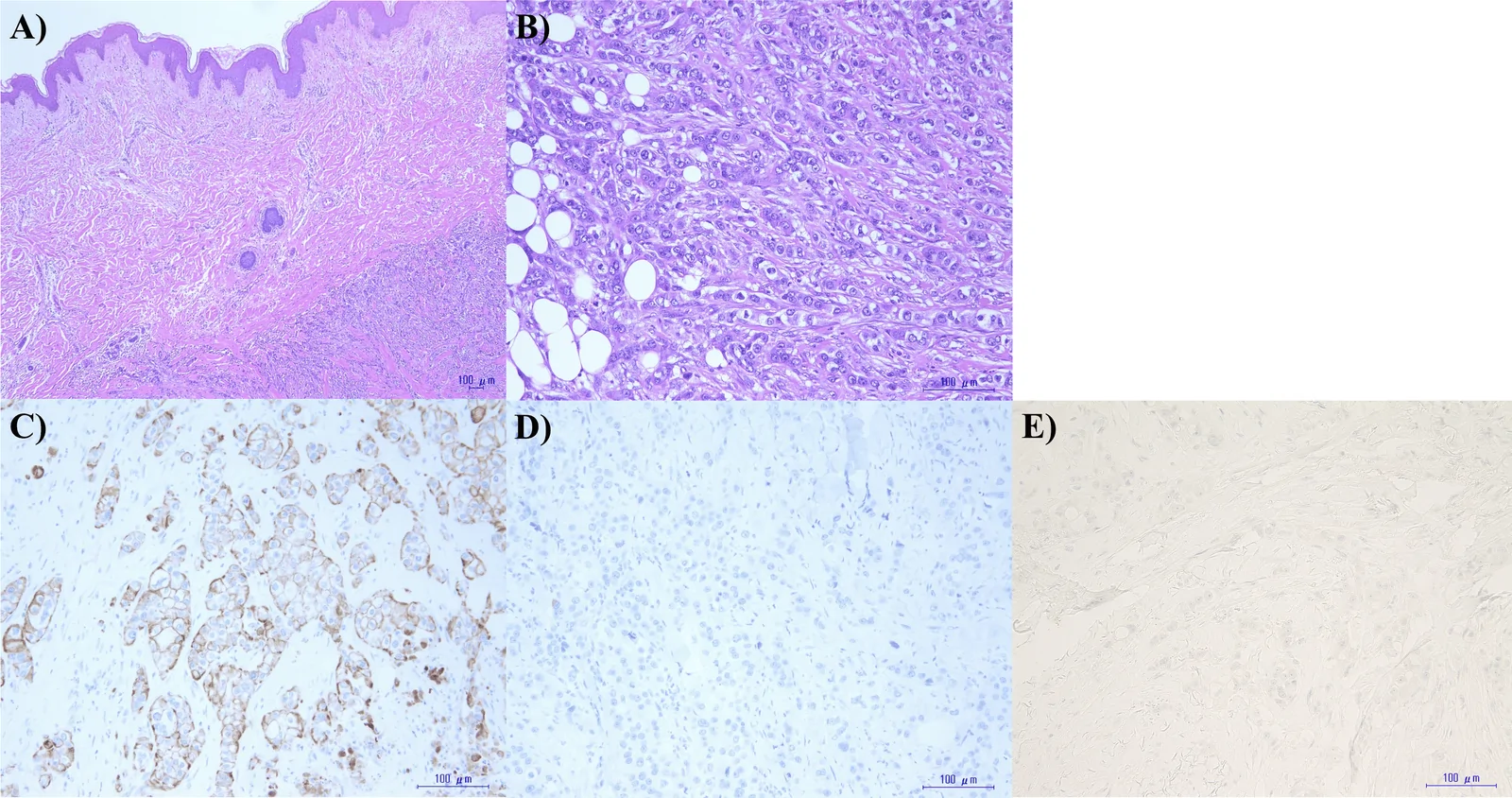
Histopathology of the cutaneous lesion. Hematoxylin and eosin (H&E) staining shows metastatic adenocarcinoma centered in the subcutaneous tissue with invasion into the dermis. Immunohistochemistry demonstrates CK7 positivity and CK20 and PAX8 negativity. (A, B) H&E, low- and high-power views; (C) CK7; (D) CK20; (E) PAX8. Original magnification: (A) ×40; (B–E) ×200.

After radiotherapy to the painful left femoral metastasis (total 30 Gy), systemic chemotherapy was initiated. Gemcitabine plus cisplatin (GC) was administered for 23 cycles, followed by 10 cycles of S‑1 (tegafur/gimeracil/oteracil potassium). During treatment, progressive disease was observed, including multiple cutaneous, bone, and lung metastases.

Best supportive care was initiated at 52 months postoperatively, and the patient died at 56 months postoperatively (Figure 5).

**Figure 5:**
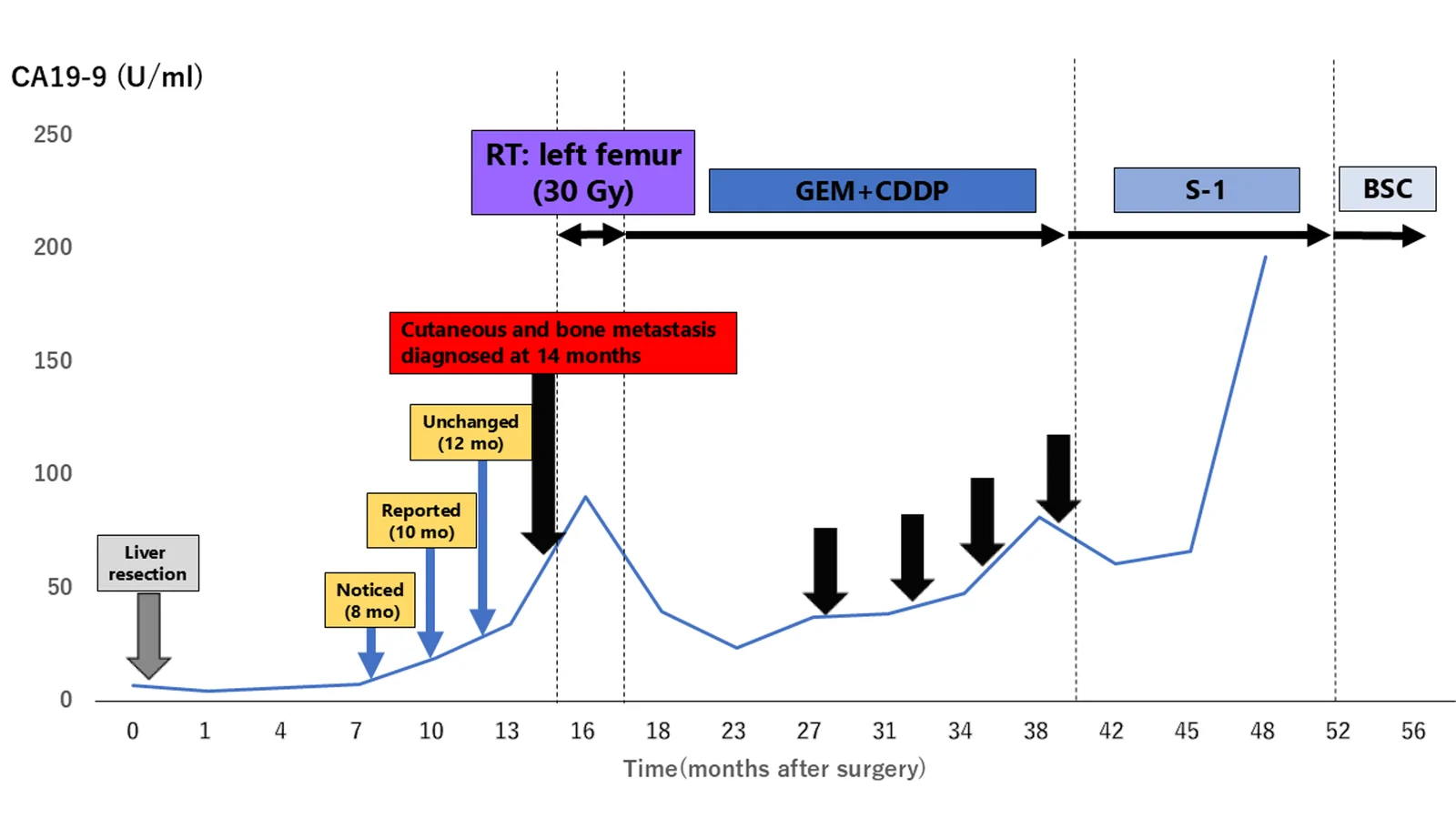
Timeline of the postoperative course, including serial CA19-9 levels, metastatic events, and treatments. Black arrows indicate excision of cutaneous metastases, and dashed lines indicate changes in systemic therapy. RT, radiotherapy; GC, gemcitabine plus cisplatin; BSC, best supportive care; S-1, tegafur/gimeracil/oteracil potassium; PO, postoperative; CA19-9, carbohydrate antigen 19-9

## Discussion

This case illustrates a rare pattern of recurrence after R0 resection of iCCA: distant cutaneous metastasis unrelated to surgical scars or drainage tracts. The postoperative subcutaneous nodule was painless, mobile, and located away from the operative field. In addition, there was no clear elevation of tumor markers and no definitive evidence of recurrence on routine imaging at the time the lesion was first reported. These factors led to an initial clinical impression of a benign lesion and delayed definitive diagnosis until pathological evaluation was performed.

Cutaneous metastasis is uncommon overall. In a retrospective study of patients with metastatic carcinoma, the most frequent primary sites were lung and breast; biliary tract cancer accounted for a small proportion of cutaneous metastases [2]. Cutaneous metastasis is often associated with disseminated disease and is generally considered to indicate poor prognosis [3].

A review of cutaneous metastases from cholangiocarcinoma found that many lesions occurred at drainage sites or surgical scars, consistent with implantation, and that approximately half of the cases were procedure-related. In addition, the median survival after diagnosis was poor, at four months (range: 21 days to 12 months) [8]. The clinical presentation is heterogeneous, including nodules, papules, or erythematous lesions, and can mimic benign dermatologic conditions [9,10]. Because biliary tract cancers are relatively uncommon and distant cutaneous metastasis is rare and variable in appearance, non-procedure-related skin lesions may be particularly challenging to recognize as recurrence during follow-up.

Postoperative surveillance for biliary tract cancer relies mainly on imaging and tumor markers [11,12]. Compared with intra-abdominal, pulmonary, or skeletal metastases, small superficial lesions may be overlooked. However, cutaneous lesions are readily accessible for biopsy, and pathological confirmation can provide an early and actionable diagnosis. In this patient, histological diagnosis of the cutaneous lesion triggered comprehensive staging and timely initiation of multimodality treatment. Despite subsequent disseminated recurrence, the patient survived for 56 months after the initial surgery.

Clinicians should maintain a low threshold for dermatologic consultation or biopsy when new postoperative subcutaneous masses arise, even when tumor markers are normal and imaging studies do not demonstrate recurrence.

## Conclusions

Cutaneous metastasis from cholangiocarcinoma can appear as distant, benign-appearing subcutaneous nodules that escape detection by routine imaging and tumor markers. This case demonstrates that even small postoperative superficial lesions may represent systemic recurrence. Pathological confirmation of such lesions can provide the first evidence of recurrence and may prompt further systemic evaluation. Early biopsy or dermatologic evaluation of new subcutaneous nodules can facilitate prompt diagnosis.

When cutaneous metastasis is confirmed, comprehensive systemic evaluation should be considered to assess for additional metastatic disease and guide subsequent treatment.
